# COVID-19 deaths on weekends

**DOI:** 10.1186/s12889-023-16451-8

**Published:** 2023-08-22

**Authors:** Fizza Manzoor, Donald A. Redelmeier

**Affiliations:** 1 Department of Medicine, University of Toronto, Toronto, Canada; 2 Institute for Clinical Evaluative Sciences, Toronto, Canada; 3 Evaluative Clinical Sciences, Sunnybrook Research Institute, Sunnybrook Hospital, G-151, 2075 Bayview Ave, ON M4N 3M5 Toronto, Canada

**Keywords:** COVID-19, Weekend mortality, Risk perception, Healthy system performance, Daily trends, Pitfalls in reasoning

## Abstract

**Background:**

Mortality statistics about daily deaths might change on weekends due to delays in reporting, uneven staffing, a different mix of personnel, or decreased efficiency. We hypothesized that reported deaths for COVID-19 might increase on weekends compared to weekdays.

**Methods:**

We collected data from the World Health Organization COVID-19 database. All deaths from March 7, 2020 to March 7, 2022 were included (two years). The primary analysis evaluated mean daily deaths on weekends compared to the preceding five workdays. Analyses were replicated in ten individual countries: United States, United Kingdom, France, Germany, Italy, Spain, Russia, India, Brazil, and Canada.

**Results:**

The mean COVID-19 daily deaths was higher on weekends compared to weekdays (8,532 vs. 8,083 *p* < 0.001), equal to a 6% relative increase (95% confidence interval 3% to 8%). The highest absolute increase was in the United States (1,483 vs. 1,220 deaths, *p* < 0.001). The second highest absolute increase was in Brazil (1,061 vs. 823 deaths, *p* < 0.001). The increase in deaths on weekends remained significant during the earlier and later months of the pandemic, as well as during the greater and lesser weeks of the pandemic.

**Conclusions:**

The apparent increased COVID-19 deaths reported on weekends might potentially reflect patient care, confound community trends, and affect the public perception of risk.

## Background

Novel coronavirus disease (COVID-19) has resulted in over 6 million deaths worldwide [1]. Daily reports of COVID-19 deaths are closely tracked by the public, political leaders, healthcare professionals, and mainstream media. Mortality counts, however, might vary on weekends due to reporting delays, fallible documentation, decreased efficiency, uneven staffing, or other factors [2, 3]. Of course, the pandemic has affected healthcare systems worldwide, and trends in weekend mortality may have changed with greater awareness, funding, and incentives [4, 5].

Some studies have explored mortality during the COVID-19 pandemic. Early studies during the pandemic have demonstrated an increase in mortality on weekdays compared to weekends [6–8]. One study evaluated behaviour change and found an increase in mobility on weekdays as a potential proxy for social distancing and predictor of mortality risk [9]. No study has examined mortality trends longitudinally or across individual countries. We explored whether COVID-19 mortality was higher on weekends compared to weekdays throughout the pandemic.

## Methods

We collected data from the World Health Organization COVID-19 database [10]. All global deaths related to COVID-19 between March 7, 2020 and March 7, 2022 were included (104 weeks). The primary analysis evaluated the mean daily deaths on weekends (Saturday and Sunday) compared to the mean deaths on the immediately preceding five workdays using a paired t-test for comparisons. Stratified analyses examined deaths among the ten individual countries with the highest COVID-19 prevalence (United States, United Kingdom, Canada, France, Germany, Italy, Spain, Russia, India, Brazil) [11]. Analyses were adjusted for national holidays and long weekends for the ten individual countries [12]. All *p*-values were two-tailed with 95% confidence intervals calculated using Microsoft Excel (version 16.66.1).

Secondary analyses evaluated the mean daily incidence of new cases reported on weekends compared to the preceding five workdays. Additional analyses also evaluated weekend mortality for earlier and later months of the pandemic (midpoint = March 7, 2021, earlier = March 2020 to March 2021, later = March 2021 to March 2022). Supplementary analyses also evaluated whether weekend mortality varied with changes in the prevalence of COVID-19 (pandemic waves) by comparing deaths during weeks with weekend mortality greater than the mean (calculated over two years) compared to weeks with weekend mortality lesser than the two-year mean. Relative changes in mortality or incidence were calculated by dividing the absolute change by total number of deaths or new cases over a specific time-period.

## Results

**Fig. 1:**
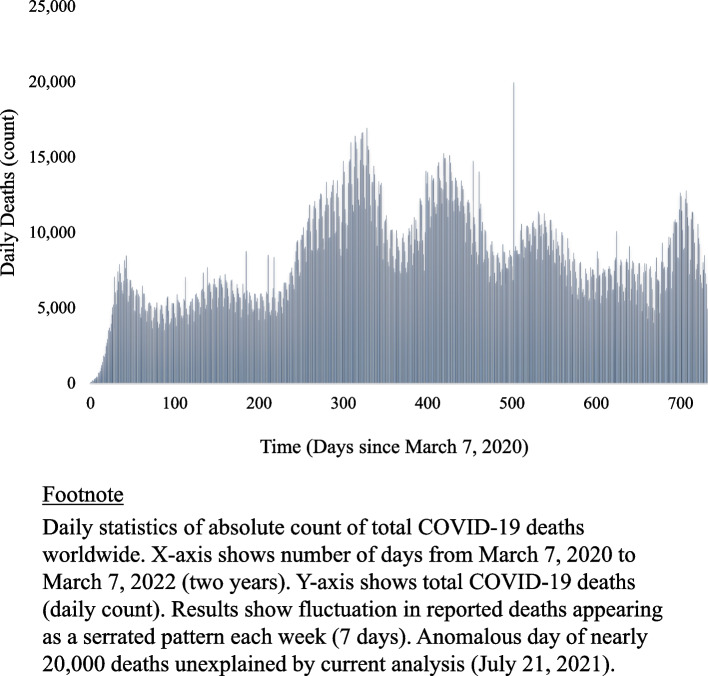
Daily deaths

A total of 5,983,471 deaths and 444,961,484 new cases were identified during the two-year interval (Fig. 1). The average number of daily global deaths from COVID-19 was higher on weekends compared to weekdays (8,532 vs. 8,083 *p* < 0.001), equal to an absolute increase of 449 deaths and a 6% relative increase (95% confidence interval 3% to 8%). The average number of incident cases was also higher on weekends compared to weekdays (646,659 vs. 594,525, *p* < 0.001), equal to an absolute increase of 52,133 cases and a 9% relative increase (95% confidence interval 1% to 17%). A concurrent apparent increase in both weekend COVID-19 mortality and incidence was apparent for 78% of weeks (81 of 104).

**Fig. 2:**
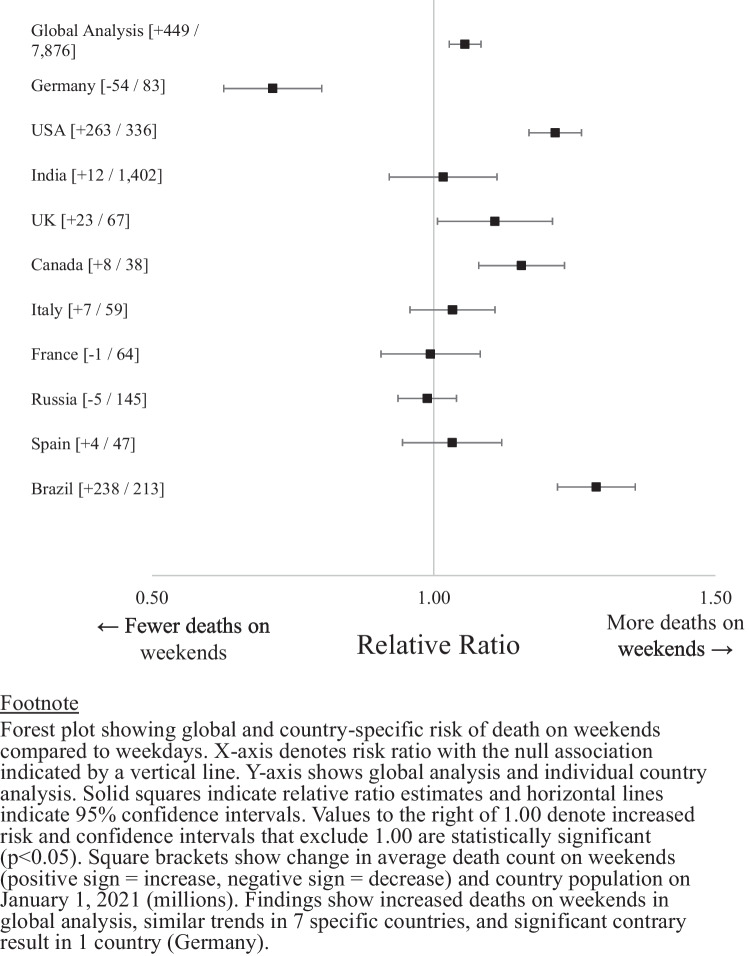
Global analysis of weekend deaths

Replication of the primary analysis among the ten individual countries (Fig. 2) showed that four countries individually had significantly higher deaths on weekends: United States (1,483 vs. 1,220 deaths, *p* < 0.001), Brazil (1,061 vs. 823 deaths, *p *< 0.001), United Kingdom (239 vs. 215 deaths, *p* < 0.001), and Canada (56 vs. 48 deaths, *p* < 0.001). Three countries showed trends towards increased deaths on weekends that were not statistically significant. Only one country showed a significant opposite pattern: Germany (134 vs. 187 deaths, *p* < 0.001).

We found the observed increase in weekend COVID-19 mortality in the global analysis persisted in the split analysis. The earlier half of the pandemic (March 2020 to March 2021) averaged 641 additional weekend deaths (7,825 vs. 7,184, *p* < 0.001) and the later half of the pandemic (March 2021 to March 2022) averaged 257 additional weekend deaths (9,239 vs. 8,982, *p* = 0.009). Of the ten countries, none had a significant contrary trend of weekend deaths over time, and all four countries with significantly more weekend deaths also showed the same trend in the later half of the study interval (United States, United Kingdom, Brazil, Canada).

We found the weekend COVID-19 mortality in the global analysis persisted regardless of overall mortality: the weeks with fewer deaths averaged 426 additional weekend deaths (6,390 vs. 5,963, *p* < 0.001) and the weeks with greater deaths averaged 476 additional weekend deaths (11,130 vs. 10,653, *p* < 0.001). Of the ten specific countries, none had a significant contrary overall trend of weekend deaths.

## Discussion

We studied nearly 6 million deaths over two years and identified a persistent global anomaly in reported COVID-19 deaths on weekends compared to weekdays. A lack of correction in the split analyses comparing earlier and later months of the pandemic further suggests this anomaly has not resolved over time. The illness course and case detection of COVID-19 does not explain variation in mortality based on the day of week [13]. We observe this anomaly in the majority of selected countries despite a diversity in public health approaches, health system structures, and funding models [14]. The main strength of our analysis is to highlight a weekly global anomaly in that COVID-19 mortality data that conflicts with the laws of nature.

Our study offers differing results compared to the current literature by showing an increase in weekend mortality during the COVID-10 pandemic [6–9]. One explanation might be delays in reporting on weekdays that are corrected on subsequent weekends. Additionally, shortfalls in staffing, hospital capacity, diagnostic services, community resources, and clinician experience may also be more common on weekends [15–18]. Furthermore, our results evaluate sustained trends throughout the pandemic that may not be identified in analyses over shorter time periods and smaller regions.

Our analysis has several limitations. We used a single database which may be limited by false negative results, missed cases, delayed updates, inconsistent taxonomy, evolving testing criteria, and data entry errors [19]. Additionally, the available data do not allow assessment of disease severity or the exact physiology underlying the increased mortality. Our analysis does not capture nuanced differences in microbiologic, systemic, and social factors affecting each separate wave of the pandemic. Although our analysis is global, the results do not explore the local policies and public health interventions in individual countries [20].

Potential solutions may consider staffing incentives, investments in community or outpatient services, public health initiatives, and weekend care model restructuring [21–23]. Furthermore, auditing database corrections of mortality counts may help identify and remedy anomalies due to reporting delays [24]. The impact of these interventions might extend beyond the COVID-19 pandemic and inform future public health response. Of course, further research is also needed to explore the specific organizational and individual factors on weekends that might increase COVID-19 mortality.

## Conclusions

Daily reports of COVID-19 deaths draw disproportionate attention to day-to-day noise, might confound projections, and potentially skew the public perception of risk [25]. The apparent increased deaths on weekends might create a false sense of security among the public on the subsequent weekdays reporting fewer deaths. Additionally, the persistently high mortality on weekends over the pandemic suggests an opportunity for improving health systems and clinical care on all days of the week. An awareness of the weekend anomaly in COVID-19 mortality might help guide policy, frame risks, and educate leaders [26, 27].

## Data Availability

Data for the study was obtained using the World Health Organization dataset (https://covid19.who.int/info).
